# Prime factorization using quantum annealing and computational algebraic geometry

**DOI:** 10.1038/srep43048

**Published:** 2017-02-21

**Authors:** Raouf Dridi, Hedayat Alghassi

**Affiliations:** 1 1QB Information Technologies (1QBit), Vancouver, British Columbia, V6C 2B5, Canada

## Abstract

We investigate prime factorization from two perspectives: quantum annealing and computational algebraic geometry, specifically Gröbner bases. We present a novel autonomous algorithm which combines the two approaches and leads to the factorization of all bi-primes up to just over 200000, the largest number factored to date using a quantum processor. We also explain how Gröbner bases can be used to reduce the degree of Hamiltonians.

Prime factorization is at the heart of secure data transmission because it is widely believed to be NP-complete. In the prime factorization problem, for a large bi-prime *M*, the task is to find the two prime factors *p* and *q* such that *M* = *pq*. In RSA cryptosystem, the message to be transmitted is encrypted using a public key which is, essentially, a large bi-prime that can only be decrypted using its prime factors, which are kept in a private key. Prime factorization also connects to many branches of mathematics; two branches relevant to us are computational algebraic geometry^1^ and quantum annealing^2^,^3^,^4^.

To leverage the problem of finding primes *p* and *q* into the realm of computational algebraic geometry, it suffices to transform it into a system of algebraic equations 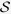. This is done using the binary representation 
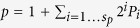
 and 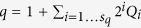, which is plugged into *M* = *pq* and expanded into a system of polynomial equations. The system 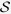 is given by this initial system of equations in addition to the auxiliary equations expressing the binary nature of the variables *P*_*i*_ and *Q*_*i*_, carry-on, and connective variables. The two primes *p* and *q* are then given by the unique zero of 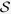. In theory, we can solve the system 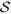 using Gröbner bases; however, in practice, this alone does not work, since Gröbner basis computation (Buchberger’s algorithm) is exponential in the number of variables.

The connection to quantum annealing can also be easily described. Indeed, finding *p* and *q* can be formulated into an unconstrained binary optimization problem 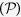, where the cost function *f* is the sum of the squares of polynomials in 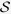. The unique zero of 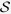 now sits on the unique global minimum of 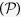 (which has minimum energy equal to zero). There are, however, a few non-trivial requirements we need to deal with before solving the cost function using quantum annealing. These requirements concern the nature of cost functions that quantum annealers can handle. In particular, we would like the cost function of 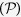 to be a positive quadratic polynomial. We also require that the coefficients of the cost function (coupling and external field parameters) be rather uniform and match the hardware-imposed dynamic range.

In the present paper, we suggest looking into the problem through both lenses, and demonstrate that indeed this approach gives better results. In our scheme, we will be using quantum annealing to solve 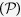, but at the same time we will be using Gröbner bases to help us reduce the cost function *f* into a positive quadratic polynomial *f*^+^ with desired values for the coefficients. We will be also using Gröbner bases at the important step of pre-processing *f*^+^ before finally passing it to the quantum annealer. This pre-processing significantly reduces the size of the problem. The result of this combined approach is an algorithm with which we have been able to factorize all bi-primes up to 2 × 10^5^ using the D-Wave 2X processor. The algorithm is autonomous in the sense that no a priori knowledge, or manual or ad hoc pre-processing, is involved. We refer the interested reader to Supplementary materials for a brief description of the D-Wave 2X processor, along with some statistics for several of the highest numbers that we embedded and solved. More detail about the processor architecture can be found in ref. 5. Another important reference is the work of S. Boixo *et al*. in ref. 6, which presents experimental evidence that the scalable D-Wave processor implements quantum annealing (with surprising robustness against noise and imperfections). Additionally, evidence that, during a critical portion of quantum annealing, the qubits become entangled and entanglement persists even as the system reaches equilibrium is presented in ref. 7.

Relevant to us also is the work in ref. 8, which uses algebraic geometry to solve optimization problems (though not specifically factorization; see *Methods* for an adaptation to factorization). Therein, Gröbner bases are used to compute standard monomials and transform the given optimization problem into an eigenvalue computation. Gröbner basis computation is the main step in this approach, which makes it inefficient. In contrast to that work, we ultimately solve the optimization problem using a quantum annealing processor and pre-process and adjust the problem with algebraic tools, that is, we reduce the size of the cost function and adjust the range of its parameters. However, we share that work’s point of view of using real algebraic geometry, and our work is the first to introduce algebraic geometry, and Gröbner bases in particular, to solve quantum annealing-related problems. We think that this is a fertile direction for both practical and theoretical endeavours.

Mapping the factorization problem into a degree-4 unconstrained binary optimization problem is first discussed in ref. 9. There, the author proposes solving the problem using a continuous optimization method he calls curvature inversion descent. Another related work is the quantum annealing factorization algorithm proposed in ref. 10. We will discuss it in the next section and improve upon it in two ways. The first involves the addition of the pre-processing stage using Gröbner bases of the cost function. This dramatically reduces the number of variables therein. The second way concerns the reduction of the initial cost function, for which we propose a general Gröbner basis scheme that precisely answers the various requirements of the cost function. In *Results*, we present our algorithm (the column algorithm) which outperforms this improved algorithm (i.e., the cell algorithm). Using a reduction proposed in ref. 10 and ad-hoc simplifications, the paper^11^ reports the factorization of bi-prime 143 on a liquid-crystal NMR quantum processor. It has been then observed by ref. 12 that the same 4-qubit Hamiltonian can be used to factor biprimes 3599, 11663, and 56153. More recently, in ref. 13, the authors factored the bi-prime 551 using a 500 MHz NMR spectrometer.

This review will not be complete without mentioning Shor’s algorithm^14^ and Kitaev’s phase estimation^15^, which, respectively, solve the factorization problem and the abelian hidden subgroup problem in polynomial time, both for the gate model paradigm. The largest number factored using a physical realization of Shor’s algorithm is 15^16^; see ref. 17 also for a discussion about oversimplification in the previous realizations. Finally, in ref. 18, it has been proved that contextuality (Kochen-Specker theorem) is needed for any speed-up in a measurement-based quantum computation factorization algorithm.

## Results

The binary multiplication of the two primes *p* and *q* can be expanded in two ways: cell-based and column-based procedures (see *Methods*). Each procedure leads to a different unconstrained binary optimization problem. The cell-based procedure creates the unconstrained binary quadratic programming problem


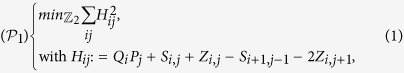


and the column-based procedure results in the problem


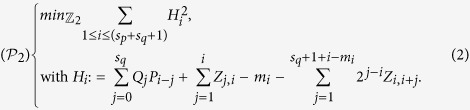


The two problems 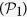 and 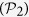 are equivalent. Their cost functions are not in quadratic form, and thus must be reduced before being solved using a quantum annealer. The reduction procedure is not a trivial task. In this paper we define, for both scenarios: (1) a reduced quadratic positive cost function and (2) a pre-processing procedure. Thus, we present two different quantum annealing-based prime factorization algorithms. The first algorithm’s decomposition method (i.e., the cell procedure) has been addressed in ref. 10, without pre-processing and without the use of Gröbner bases in the reduction step. Here, we discuss it from the Gröbner bases framework and add the important step of pre-processing. The second algorithm, however, is novel in transformation of its quartic terms to quadratic, outperforming the first algorithm due to its having fewer variables.

We write 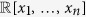 for the ring of polynomials in 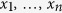 with real coefficients and 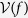 for the affine variety defined by the polynomial 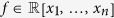, that is, the set of zeros of the equation *f* = 0. Since we are interested only in the binary zeros (i.e., 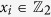), we need to add the binarization polynomials *x*_*i*_(*x*_*i*_ − 1), where *i* = 1, …, *n*, to *f* and obtain the system 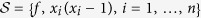. The system 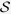 generates an ideal 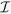 by taking all linear combinations over 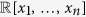 of all polynomials in 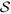; we have 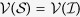. The ideal 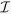 reveals the hidden polynomials which are the consequence of the generating polynomials in 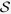. To be precise, the set of all hidden polynomials is given by the so-called radical ideal 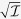, which is defined by 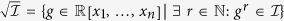. In practice, the ideal 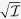 is infinite, so we represent such an ideal using a Gröbner basis 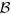 which one might take to be a triangularization of the ideal 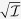. In fact, the computation of Gröbner bases generalizes Gaussian elimination in linear systems. We also have 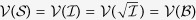 and 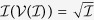. A brief review of Gröbner bases is given in *Methods*.

### The cell algorithm

Suppose we would like to define the variety 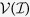 by the set of global minima of an unconstrained optimization problem 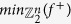, where *f*^+^ is a quadratic polynomial. For instance, we would like *f*^+^ to behave like *f*^2^. Ideally, we want *f*^+^ to remain in 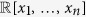 (i.e., not in a larger ring), which implies that no slack variables will be added. We also want *f*^+^ to satisfy the following requirements:

- *f*^+^ vanishes on 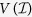 or, equivalently, 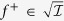.
- *f*^+^ > 0 outside 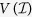, that is, *f*^+^ > 0 over 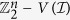.
- Coefficients of the polynomial *f*^+^ are adjusted with respect to the dynamic range allowed by the quantum processor.

Let 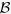 be a Gröbner basis for 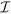. We can then go ahead and define


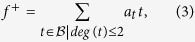


where the real coefficients *a*_*i*_ are subject to the requirements above; note that we already have 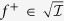 and thus the first requirement (i) is satisfied.

Let us apply this procedure to the optimization problem 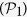 above. There, *f* = *H*_*ij*_ and the ring of polynomials is 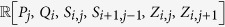. We obtain the following Gröbner basis (see *Methods* about algorithm used):


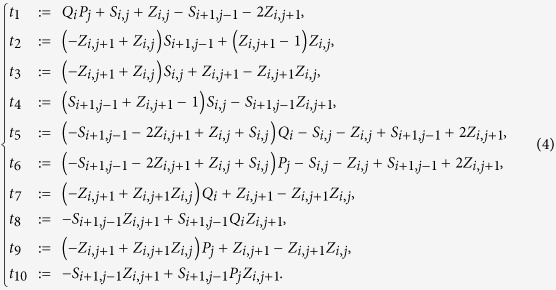


We have used the lexicographic order 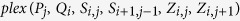; see *Methods* for definitions. Note that *t*_1_ = *H*_*ij*_. We define


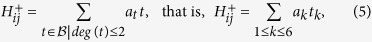


where the real coefficients *a*_*k*_ are to be found. We need to constrain the coefficients *a*_*k*_ with the other requirements. The second requirement (ii), which translates into a set of inequalities on the unknown coefficients *a*_*k*_, can be obtained through a brute force evaluation of 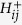 over the 2^6^ points of 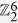. The outcome of this evaluation is a set of inequalities expressing the second requirement (ii) (see Supplementary materials).

The last requirement (iii) can be expressed in different ways. We can, for instance, require that the absolute values of the coefficients of 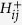, with respect to the variables *P*_*j*_, *Q*_*i*_, *S*_*i*,*j*_, *S*_*i*+1,*j*−1_, *Z*_*i*,*j*_, and *Z*_*i*,*j*+1_, be within [1 − *ε*, 1 + *ε*]. This, together with the set of inequalities from the second requirement, define a continuous optimization problem and can be easily solved. Another option is to minimize the distance between the coefficients to one specific coefficient. The different choices of the objective function and the solution of the corresponding continuous optimization problem are presented in Supplementary materials.

Having determined the quadratic polynomial 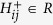 satisfyies the important requirements (i, ii, and iii) above, we can now phrase our problem 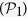 as the equivalent quadratic unconstrained binary optimization problem 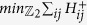. Notice that this reduction is performed only once for all cases; it need not to be redone for different bi-primes *M*. Before passing the problem to the quantum annealer, we use Gröbner bases again, this time to reduce the size of the problem. In fact, what we pass to the quantum annealer is 
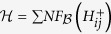
, where NF is the normal form and 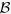 is now the Gröbner basis cutoff, which we discuss in the next section. The largest bi-prime number that we embedded and solved successfully using the cell algorithm is ~35 000. Table 1 presents a small sample of many bi-prime numbers *M* that we tested using the cell algorithm, the number of variables using both the customized reduction *CustR* (i.e., reduction explained above before pre-processing with Gröbner bases) and the window-based *GB* reduction (i.e., reduction *CustR* followed with pre-processing with Gröbner bases), the overall reduction percentage *R%*, and the embedding and solving status inside the D-Wave 2X processor *Embed*.

### The column algorithm (factoring up to 200000)

The total number of variables in the cost function of the previous method is 2*s*_*p*_*s*_*q*_, before any pre-processing. Here we present the column-based algorithm where the number of variables (before pre-processing) is bounded by 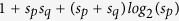. Recall that here we are phrasing the factorization problem *M* = *pq* as


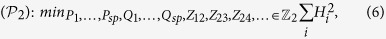


where *H*_*i*_, for 1 ≤ *i* ≤ *s*_*p*_, is


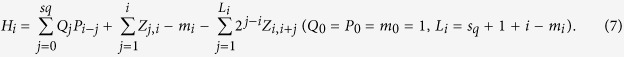


The cost function is of degree 4 and, in order to use quantum annealing, it must be replaced with a positive quadratic polynomial with the same global minimum. The idea is to replace the quadratic terms *Q*_*j*_*P*_*i*−*j*_ inside the different *H*_*i*_ with new binary variables *W*_*i*−*j*,*j*_, and add the penalty 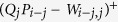 to the cost function (now written in terms of the variables *W*_*i*−*j*,*j*_). To find 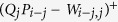, we run Gröbner bases computation on the system


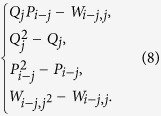


Following the same steps as in the previous section, we get


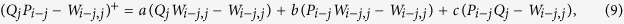


with *a, b*, 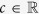 such that −*a* − *b* − *c* > 0, −*b* − *c* > 0, −*a* − *c* > 0, *c* > 0 (e.g., *c* = 1, *a* = *b* = −2). The new cost function is now


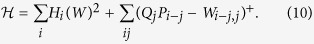


We can obtain a better Hamiltonian by pre-processing the problem before applying the *W* transformation. Indeed, let us first fix a positive integer cutoff ≤(*s*_*p*_ + *s*_*q*_ + 1) and let 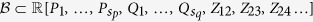 be a Gröbner basis of the set of polynomials


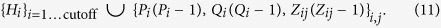


In practice, the cutoff is determined by the size of the maximum subsystem of polynomials *H*_*i*_ on which one can run a Gröbner basis computation; it is defined by the hardware. We also define a cutoff on the other tail of {*H*_*i*_}, that is, we consider 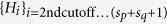. Notice that here we are working on the original *H*_*i*_ rather than the new *H*_*i*_(*W*). This is because we would like to perform the replacement 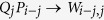 after the pre-processing (some of the quadratic terms might be simplified by this pre-processing). Precisely, what we pass to the quantum annealer is the quadratic positive polynomial


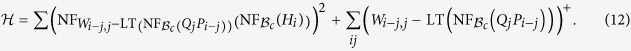


Here LT stands for the leading term with respect to the graded reverse lexicographic order. The second summation is over all *i* and *j* such that 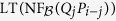 is still quadratic. The outer normal form in the first summation refers to the replacement 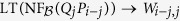, which is again performed only if 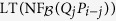 is still quadratic.

**Figure 1:**
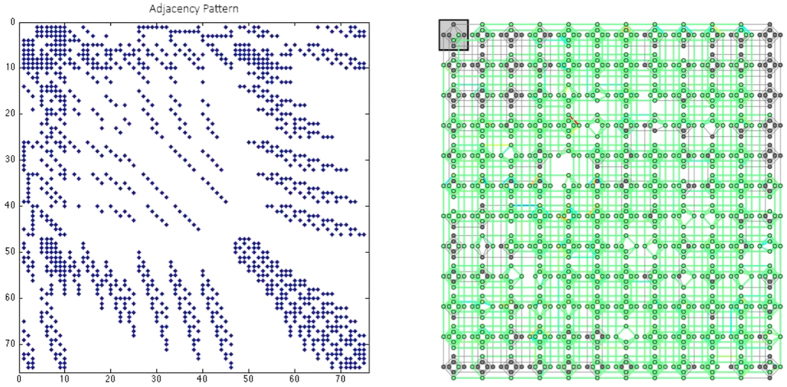
The column algorithm: the adjacency matrix pattern (left) and embedding into the the D-Wave 2X quantum processor (right) of the quadratic binary polynomial for *M* = 200099.

The columns of Table 2 present: a small sample of many bi-prime numbers that we tested and their prime factors, the number of variables using each of a naïve polynomial-to-quadratic transformation tool *P2Q* written mostly based on the algorithm discussed in ref. 19 (Other degree reduction procedures are discussed in refs 20, 21, 22, 23). Our novel polynomial-to-quadratic transformation *CustR*, and our window-based reduction *GB* after applying pre-processing. The overall reduction percentage *R%* and the embedding and solving status in the D-Wave 2X processor *Embed* are also shown. Figure 1 shows the adjacency matrix of the corresponding positive quadratic polynomial graph H and its embedded pattern inside the Chimera graph of the D-Wave 2X processor for one of the bi-primes. Details pertaining to the use of the hardware can be found in Supplementary materials.

## Discussion

In this work, factorization is connected to quantum annealing through binarization of the long multiplication. The algorithm is autonomous in the sense that no a priori knowledge, or manual or ad hoc pre-processing, is involved. We have attained the largest bi-prime factored to date using a quantum processor, though more-subtle connections might exist. A future direction that this research can take is to connect factorization (as an instance of the abelian hidden subgroup problem), through Galois correspondence, to covering spaces and thus to covering graphs and potentially to quantum annealing. We believe that more-rewarding progress can be made through the investigation of such a connection.

## Methods

### Column factoring procedure

**Figure 2:**
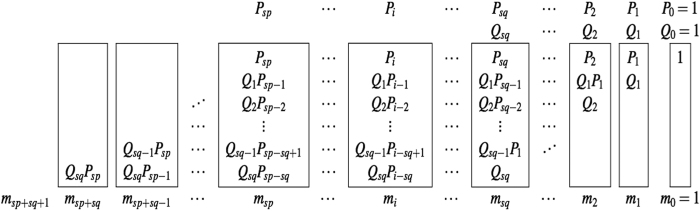
The column algorithm: the adjacency matrix pattern (left) and embedding into the the D-Wave 2X quantum processor (right) of the quadratic binary polynomial for *M* = 200099.

Here we discuss the two single-bit multiplication methods of the two primes *p* and *q*. The first method generates a Hamiltonian for each of the columns of the long multiplication expansion, while the second method generates a Hamiltonian for each of the multiplying cells in the long multiplication expansion. The column factoring procedure initially introduced in ref. 9, has been generalized. The generalized column factoring procedure of 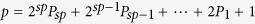 and 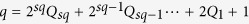 is depicted Figure 2.

The equation for an arbitrary column (*i*) can be written as the sum of the column’s multiplication terms (above) plus all previously generated carry-on terms from lower significant columns (*j* < *i*). This sum is equal to the column’s bi-prime term *m*_*i*_ plus the carry-ons generated from higher significant columns. The polynomial equation for the *i*-th column is


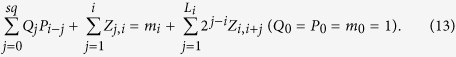


The above equation is used as the main column procedure’s equation *H*_*i*_. The Hamiltonian generation and reduction is discussed in detail in *Results*.

### Cell factoring procedure

**Figure 3:**
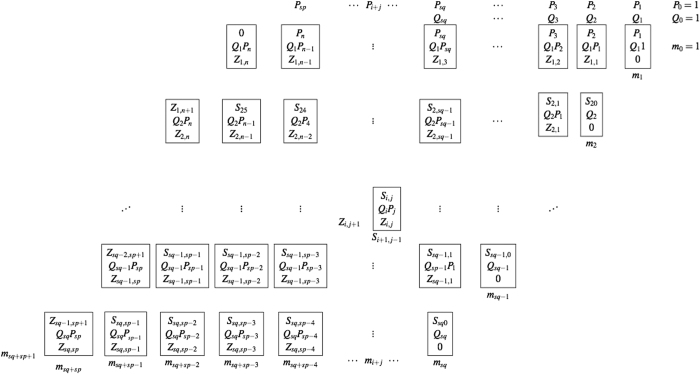
The column algorithm: the adjacency matrix pattern (left) and embedding into the the D-Wave 2X quantum processor (right) of the quadratic binary polynomial for *M* = 200099.

In the cell multiplication procedure the ultimate goal is to break each of the column equations discussed above into multiple smaller equations so that each equation contains only one quadratic term. This not only simplifies the generation of quadratic Hamiltonians, but also generates Hamiltonians with more-uniform quadratic coefficients in comparison to the column procedure. We generalized the procedure initially introduced in ref. 10. Figure 3 depicts our generalization:

Each cell contains one of the total (*s*_*p*_ − 1) (*s*_*q*_ − 1) quadratic terms in the form of *Q*_*i*_*P*_*j*_. To chain a cell to its upper cell, one extra sum variable *S*_*i*,*j*_ is added. Also, each carry-on variable *Z*_*i*,*j*_ in a cell is the carry-on of the cell directly to its right, so each cell contains four variables. The sum of three terms *Q*_*i*_*P*_*j*_, *S*_*i*,*j*_, and *Z*_*i*,*j*_ is at most 3; thus, it generates an additional sum variable *S*_*i*+1,*j*−1_ and one carry-on variable *Z*_*i*,*j*+1_. Therefore, the equation for an arbitrary cell indexed (*i, j*), shown in the centre of the above table, is


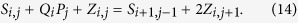


As we can see, only six binary variables are involved in each cell equation and the equation contains one quadratic term, so it can be transformed into a positive Hamiltonian without adding slack variables. The Hamiltonian generation and reduction procedure is discussed in detail in *Results*.

### Gröbner bases

Good references for the following definitions are chapters 1 and 2 of ref. 1 and chapter 1 of ref. 24.

#### Normal forms

A normal form is the remainder of Euclidean divisions in the ring of polynomials 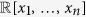. Precisely, the normal form of a polynomial 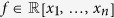, with respect to the set of polynomials 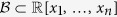 (usually a Gröbner basis), is the polynomial 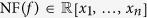, which is the image of *f* modulo 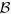. It is the remainder of the Euclidean of *f* by all 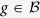.

#### Term orders

A term order on 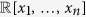 is a total order 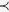 on the set of all monomials 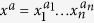, which has the following properties: (1) if 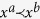, then 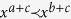 for all positive integers *a, b*, and *c*; (2) 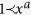 for all strictly positive integers *a*. An example of this is the pure lexicographic order 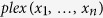. Monomials are compared first by their degree in *x*_1_, with ties broken by degree in *x*_2_, etc. This order is usually used in eliminating variables. Another example, is the graded reverse lexicographic order 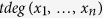. Monomials are compared first by their total degree, with ties broken by reverse lexicographic order, that is, by the smallest degree in *x*_*n*_, *x*_*n*−1_, etc.

#### Gröbner bases

Given a term order 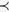 on 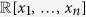, then by the leading term (initial term) LT of *f* we mean the largest monomial in *f* with respect to 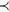. A (reduced) Gröbner basis to the ideal 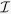 with respect to the ordering 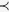 is a subset 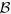 of 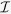 such that: (1) the initial terms of elements of 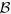 generate the ideal 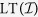 of all initial terms of 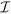; (2) for each 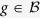, the coefficient of the initial term of *g* is 1; (3) the set LT(*g*) minimally generates 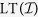; and (4) no trailing term of any 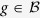 lies in 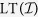. Currently, Gröbner bases are computed using sophisticated versions of the original Buchberger algorithm, for example, the F4 and F5 algorithms by J. C. Faugère^25^,^26^.

### Factorization as an eigenvalue problem

In this section, for completeness, we describe how the factorization problem can be solved using eigenvalues and eigenvectors. This is an adaptation of the method presented in ref. 8 to factorization, which is itself an adaption to real polynomial optimization of the method of solving polynomial equations using eigenvalues in ref. 1.

Let 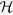 be in 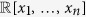 as in (12), where we have used the notation *x*_*i*_ instead of the *P*_*s*_, *Q*_*s*_, *Z*_*s*_, and *W*_*s*_. Define


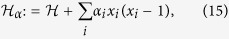


which is in the larger ring 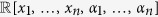. We also define the set of polynomials


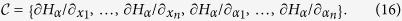


The variety 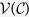 is the set of all binary critical points of 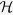. Its coordinates ring is the residue algebra 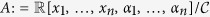. We need to compute a basis for *A*. This is done by first computing a Gröbner basis for 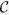 and then extracting the standard monomials (i.e., the monomials in 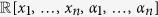 that are not divisible by the leading term of any element in the Gröbner basis). In the simple example below, we do not need to compute any Gröbner basis since 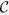 is a Gröbner basis with respect to *plex*(*α, x*). We define the linear map


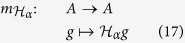


Since the number of critical points is finite, the algebra *A* is always finite-dimensional by the Finiteness Theorem (page 39 of ref. 1). Now, the key points are:

- The value of 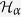 (i.e., values of 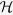), on the set of critical points 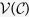, are given by the eigenvalues of the matrix 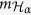.
- Eigenvalues of 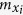 and 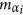 give the coordinates of the points of 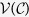.
- If *v* is an eigenvector for 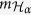, then it is also an eigenvector for 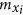 and 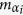 for 1 ≤ *i* ≤ *n*.

We illustrate this in an example. Consider *M* = *pq* = 5 × 3 and let


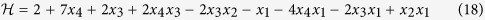


be the corresponding Hamiltonian as in (12), where *x*_1_ = *p*_2_, *x*_2_ = *q*_1_, *x*_3_ = *w*_2,1_, and *x*_4_ = *z*_2,3_. A basis for the residue algebra *A* is given by the set of the 16 monomials


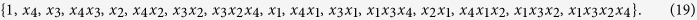


The matrix 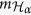 is


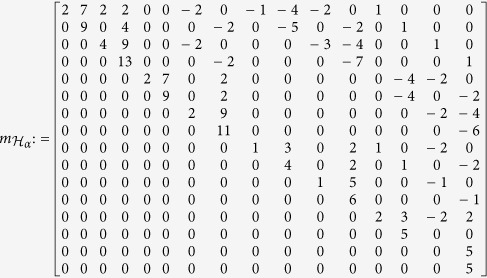


We expect the matrix’s smallest eigenvalue to be zero and, indeed, we get the following eigenvalues for 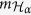:


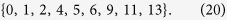


This is also the set of values which 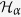 takes on 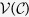. The eigenvector *v* which corresponds to the eigenvalue 0 is the column vector


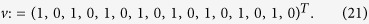


This eigenvector is used to find the coordinates of 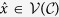 that cancel (minimize) 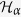. The coordinates of the global minimum 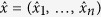 are defined by 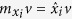, and this gives *x*_1_ = *x*_2_ = *x*_3_ = 1, *x*_4_ = 0, and *α*_1_ = 2*α*_2_ = *α*_3_ = 2, *α*_4_ = 5.

## Additional Information

**How to cite this article:** Dridi, R. and Alghassi, H. Prime factorization using quantum annealing and computational algebraic geometry. *Sci. Rep.* **7**, 43048; doi: 10.1038/srep43048 (2017).

**Publisher's note:** Springer Nature remains neutral with regard to jurisdictional claims in published maps and institutional affiliations.

## Supplementary Material

Supplementary Materials

## Figures and Tables

**Table 1:** Reduction and embedding statistics using Cell Algorithm for a sample of bi-primes.

*M*	*p* × *q*	*CustR*	*GB*	*R*%	*Embed*
31861	211 × 151	111	95	14	✓
34889	251 × 139	111	95	14	✓
46961	311 × 151	125	109	13	×
150419	431 × 349	143	125	12	×

**Table 2:** Reduction and embedding statistics using Column Algorithm for a sample of bi-primes.

*M*	*p* × *q*	*P*2*Q*	*CustR*	*GB*	*R*	*Embed*
150419	431 × 349	116	86	73	37	✓
151117	433 × 349	117	88	72	38	✓
174541	347 × 503	117	86	72	38	✓
200099	499 × 401	115	89	75	35	✓
223357	557 × 401	125	96	80	36	×
