# Erratum: Prime factorization using quantum annealing and computational algebraic geometry

**DOI:** 10.1038/srep44963

**Published:** 2017-03-22

**Authors:** Raouf Dridi, Hedayat Alghassi

Scientific Reports 7: Article number: 43048; 10.1038/srep43048 published online: 02 21 2017; updated: 03 22 2017.

In this Article, the legend of Figure 2 is incorrect:

“The column algorithm: the adjacency matrix pattern (left) and embedding into the the D-Wave 2X quantum processor (right) of the quadratic binary polynomial for M = 200099”.

Should read:

“Column factoring procedure”.

In addition, the legend of Figure 3 is incorrect:

“The column algorithm: the adjacency matrix pattern (left) and embedding into the the D-Wave 2X quantum processor (right) of the quadratic binary polynomial for M = 200099”.

Should read:

“Cell factoring procedure”.

