# Supplementary material for: Prime factorization using quantum annealing and computational algebraic geometry
Source: Sci Rep. 2017 Feb 21;7:43048. doi: 10.1038/srep43048 (PMC5318873; doi:10.1038/srep43048)
Supplement: Supplementary Materials [file srep43048-s1.pdf]

# Prime factorization using quantum annealing and computational algebraic geometry: Supplementary materials

**Raouf Dridi**[1,*] **and Hedayat Alghassi**[1,**]

[1]1QB Information Technologies (1QBit), Vancouver, British Columbia, Canada, V6C 2B5
[*]raouf.dridi@1qbit.com
[**]hedayat.alghassi@1qbit.com

## ABSTRACT

We give present 1) the embedding and solving statistics using D-Wave 2X processor DW2X_SYS4, in addition to Gröbner bases computations details 2) solutions of the continuous optimization problem introduced in *Results* and 3) a basic description of the quantum annealing processor we have used i.e., DW2X_SYS4.

## Embedding, solving and Gröbner bases computations details

We have used one of the D-Wave 2X processors, DW2X_SYS4, as our quantum annealing solver. This processor operates at a temperature range of 26(±5) millikelvin (mK) and has 1100 qubits with a 95.5-qubit yield. To utilize the processor, we used D-Wave's SAPI software development kit (version 2.2.1). To embed the problem graph into the hardware graph we used the sapiFindEmbedding and sapiEmbedProblem modules, and to solve the problems we used the sapiSolveIsing and sapiUnembedAnswer modules. For all problems we opted for the maximum number of reads available (10 000) in order to increase the fraction of ground state samples. The following table shows some statistics of the embedding and solving stages for several of the highest numbers that we were able to successfully embed and solve.

*Embedding & Solving Statistics*

| $M$ | $n$ | $emTry$ | $idC$ | $prC$ | $\#qubits$ | $jRatio$ | $rTime$ |
|---|---|---|---|---|---|---|---|
| 31861 | 95 | 33 | 848 | 721 | 815 | 10 | 3.52 |
| 34889 | 95 | 27 | 803 | 740 | 833 | 10 | 3.52 |
| 150419 | 73 | 1 | 941 | 830 | 902 | 64 | 3.52 |
| 151117 | 72 | 7 | 1001 | 846 | 918 | 64 | 3.52 |
| 174541 | 72 | 3 | 1004 | 897 | 966 | 64 | 3.52 |
| 200099 | 75 | 5 | 884 | 824 | 897 | 64 | 3.52 |

In the above table, $M$ stands for the bi-prime, $n$ is the number of variables in the QUBO problem, *emTry* is the number of block trials of the sapiFindEmbedding routine, *idC* is the total number of identified couplers, *prC* is the total number of problem couplers, *#qubits* is the total number of (physical) qubits, *jRatio* is the ratio $\frac{\max(\{|J_{ij}|\})}{\min(\{|J_{ij}|\})}$, and *rTime* is the chip run time in seconds.

For Gröbner bases computation, we have used J. C. Faugère *FGb* C library available at http://www-polsys.lip6.fr/~jcf/. The calculations were performed on 2.5 GHz Intel Core i5 processor with 16 GB RAM. This hardware constrained the cutoff to be around 8 and around 4 for $(s_p + s_q + 1) - 2\mathrm{ndcutoff}$. The percentage $R\%$ of the obtained reduction is reported in the main text (Cell Algorithm and Column Algorithm tables) and is around 13% for the Cell Algorithm and 35% for the Column Algorithm. We could not achieve more reduction (for the bi-prime numbers reported) using this hardware.

## Continuous optimization problems for the requirements (ii–iii)

In *Results*, we describe how a positive quadratic polynomial $H^+_{ij}$ can be extracted using Gröbner bases. Here we provide the details of the calculation.

The second requirement (ii) is equivalent to each of the following linear polynomials being greater than zero:

$$a_1, -a_1+a_3, -a_1-a_4, -a_1+a_5, -a_1+a_6, 2a_1+a_3, 2a_1-a_4, -a_2-a_1, -a_2+2a_1, -2a_1+a_3+2a_5, -2a_1+a_3+2a_6, -2a_1-a_4+2a_5, -2a_1-a_4+2a_6, -a_1+a_5+a_6, a_1+a_3-a_5, a_1+a_3-a_6, a_1-a_4-a_5, a_1-a_4-a_6, -a_2-2a_1+2a_5, -a_2-2a_1+2a_6, -a_2+a_1-a_5, -a_2+a_1-a_6, -2a_1+a_3+2a_5+2a_6, -2a_1-a_4+2a_5+2a_6, a_1+a_3-a_5-a_6, a_1-a_4-a_5-a_6, -a_2-2a_1+a_3-a_4, -a_2-2a_1+2a_5+2a_6, -a_2+a_1-a_5-a_6, -a_2+3a_1+a_3-a_4, -a_2-3a_1+a_3-a_4+3a_5, -a_2-3a_1+a_3-a_4+3a_6, -a_2+2a_1+a_3-a_4-2a_5, -a_2+2a_1+a_3-a_4-2a_6, -a_2-3a_1+a_3-a_4+3a_5+3a_6, -a_2+2a_1+a_3-a_4-2a_5-2a_6$$

For the third requirement (iii), the first choice for the objective function $f:\mathbb{R}^5 \to \mathbb{R}$ is

$$\begin{aligned} f(a_1,\ldots,a_6) &= \left((-a_1+a_5+a_6)^2-1\right)^2+\left((-2a_1+a_3+2a_5+2a_6)^2-1\right)^2 \\ &+ \left((a_1-a_2-a_5-a_6)^2-1\right)^2+\left((a_1-a_4-a_5-a_6)^2-1\right)^2 \\ &+ 2\left({a_2}^2-1\right)^2+\left({a_1}^2-1\right)^2+2\left({a_3}^2-1\right)^2+2\left({a_4}^2-1\right)^2 \\ &+ 2\left({a_5}^2-1\right)^2+2\left({a_6}^2-1\right)^2+\left(4{a_5}^2-1\right)^2+\left(4{a_6}^2-1\right)^2 \end{aligned}$$

The solution is $a_1=0.214$, $a_2=-1.082, a_3=0.514, a_4=-1.082$, $a_5=0.314$, and $a_6=0.314$.

The second choice for $f$ is

$$\begin{aligned} f(a_1,\ldots,a_6) &= \left((-a_1+a_5+a_6)^2-a_2\right)^2+\left((-2a_1+a_3+2a_5+2a_6)^2-a_2\right)^2 \\ &+ \left((a_1-a_2-a_5-a_6)^2-a_2\right)^2+\left((a_1-a_4-a_5-a_6)^2-a_2\right)^2 \\ &+ 2\left({a_2}^2-a_2\right)^2+\left({a_1}^2-a_2\right)^2+2\left({a_3}^2-a_2\right)^2+2\left({a_4}^2-a_2\right)^2 \\ &+ 2\left({a_5}^2-a_2\right)^2+2\left({a_6}^2-a_2\right)^2+\left(4{a_5}^2-a_2\right)^2+\left(4{a_6}^2-a_2\right)^2 \end{aligned} \tag{1}$$

The solution is $a_1=1.0, a_2=-4.0, a_3=4.0, a_4=-4.0, a_5=2.0$, and $a_6=2.0$ (identical to the solution given in[1]).

## Basic description of the quantum annealing processor

Here we introduce the quantum annealing concept that ultimately solves a general Ising (quadratic unconstrained binary optimization, or "QUBO") problem, then talk about the important topic of embedding a QUBO problem into the specific quantum annealer (the D-Wave 2X processor).

Quantum annealing (QA), along with the D-Wave processors, have been the focus of much research. We refer the interested reader to[2–9]. QA is a paradigm designed to find the ground state of systems of interacting spins represented by a time-evolving Hamiltonian:

$$\mathscr{S}(s)=\mathscr{E}(s)\mathscr{H}_P-\frac{1}{2}\sum_i \Delta(s)\sigma_i^x,$$

$$\mathscr{H}_P=-\sum_i h_i\sigma_i^x+\sum_{i<j} J_{ij}\sigma_i^z\sigma_j^z.$$

The parameters $h_i$ and $J_{ij}$ encode the particular QUBO problem $P$ into its Ising formulation. QA is performed by first setting $\Delta \gg \mathscr{E}$, which results in a ground state into which the spins can be easily initialized. Then $\Delta$ is *slowly* reduced and $\mathscr{E}$ is increased until $\mathscr{E} \gg \Delta$. At this point the system is dominated by $\mathscr{H}_P$, which encodes the optimization problem. Thus, the ground state represents the solution to the optimization problem.

An embedding is the mapping of the nodes of an input graph to the nodes of the destination graph. The graph representing the problem's QUBO matrix needs to be embedded into the actual physical qubits on the processor in order for it to solve

the QUBO problem. The specific existing connectivity pattern of qubits in the D-Wave chip is called the Chimera graph. Embedding an input graph (a QUBO problem graph) into the hardware graph (the Chimera graph) is in general NP-hard ([10]).

Figure 1–right shows an embedding of the (column algorithm) QUBO corresponding to the bi-prime $M = 200\,099$ into the Chimera graph of the D-Wave 2X chip consisting of a 12 by 12 lattice of 4 by 4 bipartite blocks. The Chimera graph is structured so that the vertical and horizontal couplers in its lattice are connected only to either side of each bipartite block. Each node in this graph represents one qubit and each edge represents a coupling between two qubits. Adjacent nodes in the Chimera graph can be grouped together to form new effective (i.e., logical) nodes, creating nodes of a higher degree. Such a grouping is performed on the processor by setting the coupler between two qubits to a large negative value, forcing two Ising spins to align such that the two qubits end up with the same values. These effective qubits are expected to behave identically and remain in the same binary state at the time of measurement. The act of grouping adjacent qubits (hence forming new effective qubits) is called chain creation or chain identification.

An embedding strategy consists of two tasks: mapping and identification. Mapping is the assignment of the nodes of the input graph to the single or effective nodes of the destination graph. Solving such problems optimally is in general NP-hard, but one can devise various approximations and enhancement strategies to overcome these difficulties, for example, using statistical search methods like simulated annealing, structure-based methods, or a combination of both. For a better understanding of current embedding approaches, we refer the reader to[10–13]. In Figure 1–right, the blue lines indicate the identified couplers, the yellow lines indicate the problem couplers (i.e., the edges of the problem graph), and the grey lines indicate empty couplers.

## References


1. Schaller, G. & Schutzhold, R. The role of symmetries in adiabatic quantum algorithms. *Quantum Information & Computation* **10**, 109–140 (2010).
2. Kadowaki, T. & Nishimori, H. Quantum annealing in the transverse ising model. *Phys. Rev. E* **58**, 5355–5363 (1998).
3. Farhi, E. *et al.* A quantum adiabatic evolution algorithm applied to random instances of an np-complete problem. *Science* **292**, 472–475 (2001).
4. Das, A. & Chakrabarti, B. K. *Colloquium* : Quantum annealing and analog quantum computation. *Rev. Mod. Phys.* **80**, 1061–1081 (2008).
5. Johnson, M. W. *et al.* Quantum annealing with manufactured spins. *Nature* **473**, 194–198 (2011).
6. Calude, C. S., Calude, E. & Dinneen, M. J. Guest column: Adiabatic quantum computing challenges. *SIGACT News* **46**, 40–61 (2015).
7. Boixo, S., Albash, T., Spedalieri, F. M., Chancellor, N. & Lidar, D. A. Experimental signature of programmable quantum annealing. *Nat Commun* **4** (2013).
8. Boixo, S. *et al.* Evidence for quantum annealing with more than one hundred qubits. *Nat Phys* **10**, 218–224 (2014).
9. Lanting, T. *et al.* Entanglement in a quantum annealing processor. *Phys. Rev. X* **4**, 021041 (2014).
10. Choi, V. Minor-embedding in adiabatic quantum computation: I. the parameter setting problem. *Quantum Information Processing* **7**, 193–209 (2008).
11. Bian, Z. *et al.* Discrete optimization using quantum annealing on sparse ising models. *Frontiers in Physics* **2** (2014).
12. Jun, C., William, G. M. & Aidan, R. A practical heuristic for finding graph minors. *Preprint arXiv:1406.2741* (2014).
13. Tomas, B., Andrew, D. K. & Aidan, R. Fast clique minor generation in chimera qubit connectivity graphs. *Preprint arXiv:1507.04774* (2015).
